# Case report of severe bronchial web-like stenoses after ‘surviving the unsurvivable’

**DOI:** 10.1186/s12890-019-0873-z

**Published:** 2019-07-02

**Authors:** Thomas Crowhurst, Joshua Lightfoot, Aeneas Yeo, Benjamin Reddi, Phan Nguyen, Helen Whitford, Chien-Li Holmes-Liew

**Affiliations:** 1 Discipline of Medicine, University of Adelaide, Adelaide, SA 5000 Australia; 2 Department of Thoracic Medicine, Central Adelaide Local Health Network, Royal Adelaide Hospital, 1 Port Road, Adelaide, SA 5000 Australia; 3 Intensive Care Unit, Central Adelaide Local Health Network, Royal Adelaide Hospital, 1 Port Road, Adelaide, SA 5000 Australia; 4 Discipline of Acute Care Medicine, University of Adelaide, Adelaide, SA 5000 Australia; 5 Department of Respiratory Medicine, The Alfred Hospital, 55 Commercial Road, Melbourne, Victoria 3004 Australia; 6 Department of Medicine, Monash University, Wellington Road, Clayton, Victoria 3800 Australia

**Keywords:** Inhalation injury, Extra-corporeal membrane oxygenation, Bronchial web-like stenoses, Interventional pulmonology, Lung transplantation

## Abstract

**Background:**

There are few cases of multiple bronchial stenoses reported in the literature and none of the severity described here. The case is relevant due to its rareness, the pathophysiological insights derived, the successful interventional pulmonology strategies demonstrated, and as an example of a rare indication for high-risk lung transplantation.

**Case presentation:**

A 47-year-old man developed multiple recurrent bronchial web-like stenoses five weeks after an episode of severe tracheo-bronchitis presumed secondary to a chemical inhalation injury which initially caused complete bilateral lung collapse necessitating veno-venous extracorporeal membrane oxygenation. The stenoses completely effaced bronchi in many locations causing severe type II respiratory failure requiring mechanical ventilation and bronchoscopic puncture / dilatation then ultimately bilateral lung transplantation.

**Conclusion:**

This very rare case highlights the morbid sequelae that can arise after catastrophic tracheobronchitis which now, in the era of extracorporeal membrane oxygenation, may be survivable in the short-term.

## Background

Bronchial stenoses are commonly caused by malignancy, trauma, infection (e.g. tuberculosis) and autoimmune conditions however they are generally singular or few [1–3]. There are very few reports of multiple airway stenoses through inhalational injury [4]. This case report seeks to draw attention to a rare case of multiple recurrent bronchial stenoses causing severe respiratory failure, successfully managed only through aggressive interventional pulmonary strategies and ultimately bilateral lung transplantation.

**Fig. 1:**
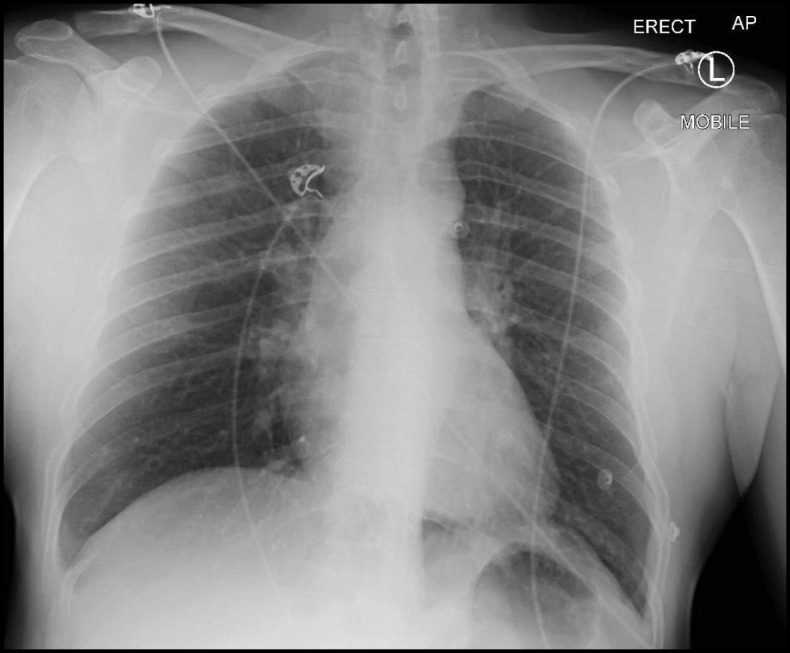
Effectively clear plain erect anteroposterior chest x-ray at presentation

**Fig. 2:**
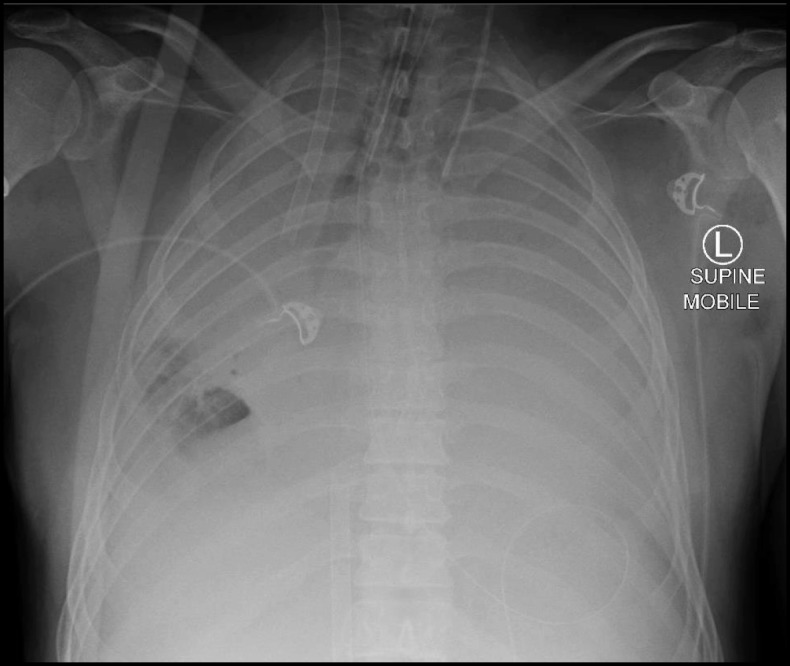
Interval plain supine anteroposterior chest x-ray demonstrating complete bilateral lung collapse three days after presentation and one day after the commencement of extra-corporeal membrane oxygenation

**Fig. 3:**
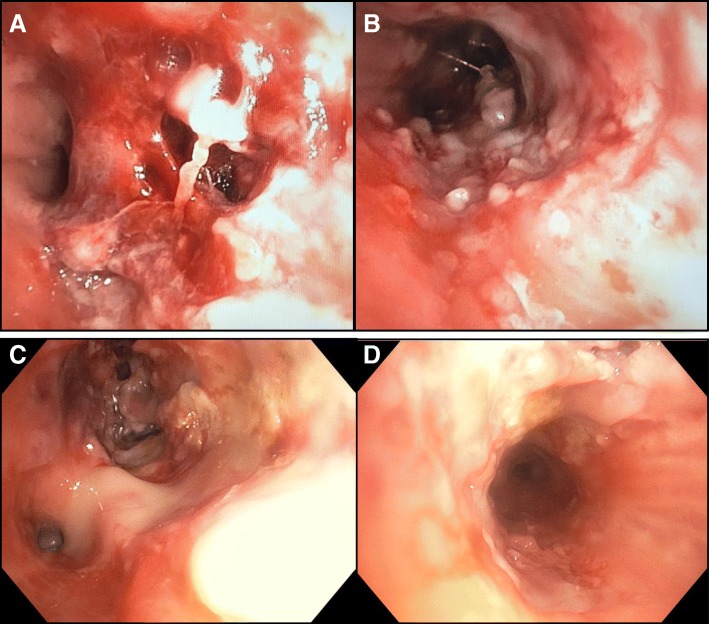
Four images of central airways from bronchoscopy performed during initial admission, revealing severe diffuse airway inflammation characterised by purulent exudate and ulcers plus nodules throughout the airways. Image **a** is from proximal left upper lobe bronchus. Image **b** is from trachea. Image **c** shows left lower lobe bronchus on the bottom left and left upper lobe bronchus on the upper middle portion. Image **d** is from right main bronchus

**Fig. 4:**
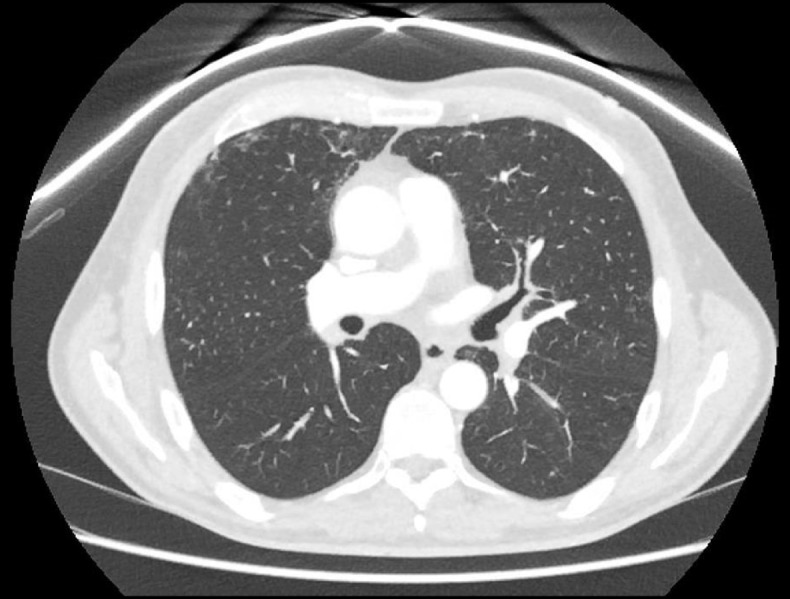
Slice from computed tomography imaging of the chest at re-presentation demonstrating bronchial wall thickening, in this instance particularly notable in the left upper lobe bronchus and proximal portion of the left anterior segment bronchus (Lb3)

**Fig. 5:**
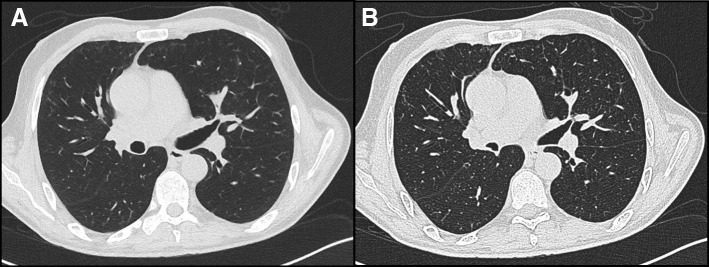
Two slices from computed tomography imaging of the chest approximately three weeks after re-presentation demonstrating with image **a** on the left performed at full inspiration and image **b** on the right performed at full expiration. The images demonstrate some mild improvement in the bronchial wall thickening affecting the left upper lobe bronchus seen in image 4 but, more importantly, little change in lung lucency on expiration due to significant homogenous gas trapping

**Fig. 6:**
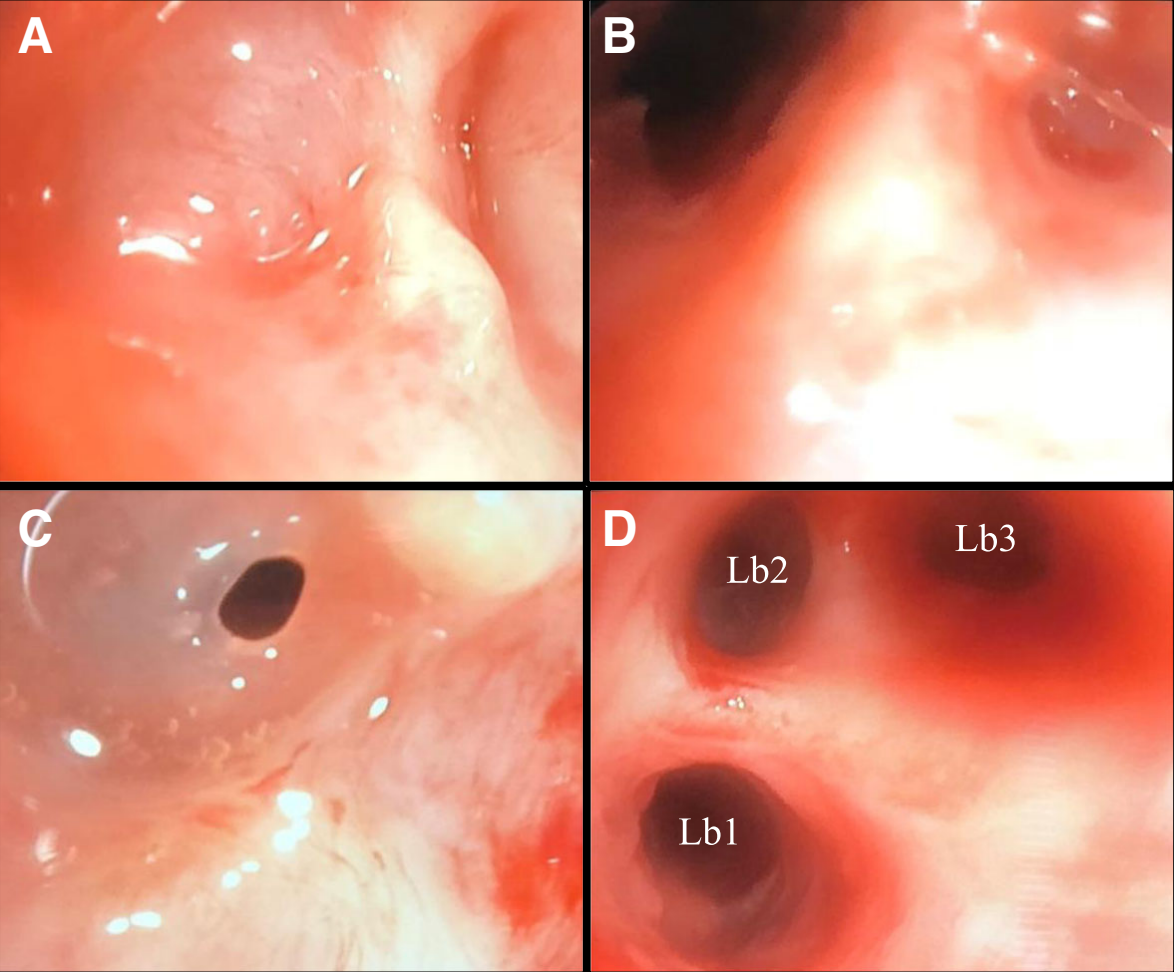
Bronchoscopic images after deterioration with severe type II respiratory failure requiring intubation and mechanical ventilation. Image **a** demonstrates total membranous occlusion of Rb1b and image **b** demonstrates this airway after opening via puncture of the membranous stenosis by Wang needle then balloon dilatation. Image **c** reveals near complete stenosis of the left upper lobe superior division bronchus. Image **d** demonstrates the view beyond this stenosis after dilatation, revealing a normal variant Lb1 / Lb2 / Lb3 configuration of the left upper lobe segmental bronchi

## Case presentation

A 47-year-old previously well male electrician from rural Australia presented with five days of worsening dyspnoea, productive cough and scant haemoptysis unresponsive to oral antibiotics and corticosteroids. He had a history of depression (desvenlafaxine) and active tobacco smoking (25 pack years). Initial chest x-ray was normal. He rapidly progressed to severe type I respiratory failure over the ensuing day requiring intubation and mechanical ventilation. Marked inspiratory and expiratory airflow limitation precluded adequate gas exchange and therefore veno-venous extra-corporeal membrane oxygenation (ECMO) was emergently instituted. Complete bilateral lung collapse developed over the next day (Figs. 1 and 2).

The lack of airspace opacity on initial chest x-ray excluded acute respiratory distress syndrome and suggested the severe airflow limitation may be due to airway obstruction. Bronchoscopy confirmed severe diffuse airway inflammation characterised by purulent exudate, ulcers and nodules throughout the airways (Fig. 3). Endobronchial biopsies revealed an acute necro-inflammatory process. Extensive microbiologic investigations were negative except for *Rhinovirus* identified by polymerase chain reaction. Vasculitis screen including antineutrophil cytoplasmic antibody (ANCA) was negative. The presumptive diagnosis was an inhalational injury.

Management consisted of broad-spectrum antimicrobial therapy (including meropenem, vancomycin, doxycycline and voriconazole), repeat bronchoscopic toilet and supportive care. The airway inflammation improved and transition to mechanical ventilation occurred after 13 days when only mild expiratory airflow obstruction was observed. A tracheostomy tube was sited two days after cessation of ECMO and ventilatory support was gradually weaned, ceasing seven days later. The patient was transferred to the ward on day 24 of admission. The only complication was critical-illness myopathy. He was discharged three days later for outpatient follow-up. He could mobilise 50 m and had no symptoms at rest.

The patient re-presented six days post-discharge with progressive dyspnoea, wheeze and a mild cough productive of yellow sputum. Examination revealed increased work of breathing and a diffuse wheeze throughout the respiratory cycle. Fibre-optic nasoendoscopy to mid-trachea did not reveal paradoxical vocal cord motion or obstruction. Computed tomography (CT) imaging demonstrated widespread bronchial wall thickening from large to medium airways and mild ground-glass opacity in the peripheral upper lobes bilaterally (Fig. 4). Bronchoscopy showed persistent diffuse patchy mucus coating the airways, worst in the left upper lobe where some mild narrowing was observed, however overall appearances were vastly improved compared with those during the initial admission; washings detected scant inflammatory cells, predominantly neutrophils, and no microbes.

The patient was treated empirically with high-dose prednisolone, inhaled bronchodilators and antibiotics. He had significant anxiety and was managed with sertraline and olanzapine plus pro re nata clonazepam. His dyspnoea worsened and over the ensuing 17 days he developed increasing headache and drowsiness. He was found to be in severe but partially compensated type II respiratory failure with a normal alveolar arterial oxygen (Aa) gradient (arterial blood gas on FiO_2_ 0.27 revealed pH 7.33, PaO_2_ 99 mmHg, PaCO_2_ 74 mmHg, bicarbonate 34 mmol/l).

Repeat CT imaging showed persistent but improved bronchial wall thickening but now significant homogeneous gas trapping (Fig. 5); this was thought to reflect bronchiolitis secondary to the presumed initial inhalational injury. Other differential diagnoses considered were a central deficit (neurological examination and magnetic resonance imaging of brain were normal), medications (cessation of benzodiazepines led to no benefit) and neuromuscular weakness (respiratory effort appeared significant with costal indrawing / paradoxical abdominal movements, anti-acetylcholine receptor antibodies were absent and electromyography plus nerve conduction studies were normal).

The patient deteriorated despite a trial of non-invasive ventilation, becoming obtunded (PaCO_2_ 175 mmHg) and requiring intubation and mechanical ventilation. There was difficulty with mechanical ventilation with peak inspiratory pressures of 69 cmH_2_O required to achieve tidal volumes of 3 ml/kg. Expiratory airflow limitation plus plateau airway pressures < 20 cmH_2_O indicated a predominantly obstructive ventilatory defect and intermittent circuit disconnection was required to relieve gas trapping.

Bronchoscopy during mechanical ventilation revealed multiple concentric fibrous web-like stenoses in lobar and segmental bronchi throughout both lungs. Some webs had totally effaced bronchi. Many stenoses were successfully dilated by balloon and, where membranes had effaced airways, these were punctured by Wang needle then dilated (Fig. 6). Stent placement was not feasible given the great multiplicity of stenoses and their involvement of non-central airways. Immediately post-procedure a dramatic improvement in ventilatory performance occurred. Peak inspiratory pressures dropped to 18 cmH_2_O and the patient was extubated the next day with negligible supplementary oxygen requirement. Endobronchial biopsies revealed non-specific mucosal ulceration and chronic inflammation with stromal fibrosis. Pulsed high-dose methylprednisolone was trialled for what was considered an intense proliferative / fibrotic inflammatory process arising from disordered mucosal healing secondary to a presumed inhalational injury.

The patient improved and was discharged with a plan for outpatient rehabilitation followed by repeat bronchoscopy in three weeks. Spirometry at discharge demonstrated moderate to severe obstructive pathophysiology and reduced diffusing capacity (FEV1 1.79 l [49% predicted], FVC 3.26 l [70% predicted], diffusing capacity of carbon monoxide corrected for haemoglobin 19.2 ml/min/mmHg [66% predicted]). Claustrophobia prevented plethysmography.

The patient re-presented after 6 days with increasing dyspnoea, wheeze and mild type I respiratory failure. Bronchoscopy revealed aggressive recurrence of the webbing with occlusion of various segmental bronchi, some of which were re-canalised with puncture and dilatation. Mucosa in some areas sheared away from the underlying bronchial cartilage, precluding further intervention. The trachea appeared largely spared and the proximal main bronchi only minimally affected. An extensive history was conducted including from collateral sources regarding possible inhaled exposures in his home due to the repeated deterioration early after discharge. No causative agent could be identified except for malathion which the patient had used to spray ants a few days prior to his first presentation and not since. As the exposure was only identified at this later stage, cholinesterase levels could not be performed on blood samples from the original admission.

Due to the severe and rapidly recurring webbing plus the increasing danger of endobronchial intervention, the patient was rapidly worked-up for lung transplantation. There were concerns regarding transplanting for a respiratory disease of unknown origin, that the underlying disease may be systemic, that it may affect the anastomoses and native large airways or that it may recur in the graft. Furthermore the patient was deconditioned and could not complete standard lung transplantation work-up. Surgical options included a standard bilateral sequential lung transplant versus a domino heart-lung transplant with a tracheal anastomosis. The patient underwent the former one month later at the quaternary referral centre with pathology of the explant revealing very severe necrotising bronchitis extending deep into the wall with associated granulation and fibrosis, most severe in the central bronchi, with some areas of histiocytic inflammation particularly in the distal trachea; however no specific diagnosis could be reached. Twelve months post-transplant, the patient is progressing well with normal graft function and no evidence of disease recurrence or systemic illness.

## Discussion and conclusions

This case warrants discussion for the diagnostic lessons arising from the re-presentation with type II respiratory failure, as an example of interventional pulmonology strategies, as a very rare case of severe diffuse bronchial stenoses after presumed inhalational airway injury and as an extremely rare indication for high-risk lung transplantation.

The cause of the severe type II respiratory failure upon re-presentation was initially uncertain. Differential diagnoses included parenchymal lung disease (however Aa gradient was normal and imaging not supportive) and other possibilities that were excluded as above (central nervous system disease, neuromuscular disease and drugs). Given the homogenous gas trapping on imaging, it was theorised that an initial inhalational chemical injury had caused bronchiolitis obliterans.

The discovery of severe diffuse bronchial stenoses on repeat bronchoscopy only 17 days later provided a superior explanation for the type II respiratory failure and difficulty in ventilation. This was confirmed by the dramatic improvement after bronchoscopic dilatation. We deduce collateral ventilation allowed segments distal to complete membranous bronchial occlusion to remain aerated. Fixed large airway obstruction and attenuated airflow through pathways of collateral ventilation would have retarded both inspiration, manifesting as high peak inspiratory pressure, as well as expiration, promoting the gas trapping and hypoventilation that characterised this case.

There are many potential causes of one or a few bronchial stenoses. Reported aetiologies include congenital lesions, infection (mycobacteria, diphtheria, influenza, papillomatosis, rhinoscleroma, fungal), autoimmune disorders (granulomatosis with polyangiitis, microscopic polyangiitis, sarcoidosis, Crohn’s disease, Behçet’s disease, relapsing polychondritis), inhalational injury (thermal burns, mustard gas, hydrochloric acid), benign and malignant tumours, malignancy treatment (radiotherapy, endobronchial brachytherapy), trauma (prolonged intubation, post-surgical) and miscellaneous (amyloidosis, tracheobronchopathia osteochrondoplastica, post-transplant, idiopathic) [2–6].

Far fewer causes of multiple bronchial stenoses have been identified and reports in the literature are rare. Mailloux and colleagues report a case of multiple web-like bronchial stenoses arising after severe illness secondary to infection with H1N1 influenza A which has some notable similarities with our case, including the delayed presentation after the initial airway injury [1]. Rubin et al. describe a case of multiple stenoses affecting the trachea and main / lobar bronchi which also arose one month after an initial airway injury, in their case due to inhalation of hydrochloric acid [4]. Keating and colleagues report a cause of multiple bronchial webs arising two years after lung transplantation with no cause identified [6]. Ulloa-Clavijo et al. describe a case of web-like bronchial stenoses affecting an adolescent with granulomatosis with polyangiitis [3]. Mizushima et al. report a case of multiple lobar bronchial stenoses due to sarcoidosis [7]. As in our case, many of these reports describe a significant delay in the identification of the bronchial stenoses while treatment was rendered for presumed asthma or chronic obstructive pulmonary disease; this highlights the diagnostic challenge posed by this rare phenomenon and demonstrates the superiority of bronchoscopy over CT imaging for the assessment of airway stenoses, particularly those with web-like configurations which can be narrower than CT slices yet still cause severe airway obstruction.

Malathion is an organophosphate pesticide considered to have low acute toxicity. Its effects on humans are mediated by inhibition of acetylcholinesterase, accumulation of acetylcholine and cholinergic toxicity. Malathion was the only inhaled toxin identified in our patient but the clinical features we observed are not typical of those described in historical studies of malathion toxicity in humans [8]. Although this case could potentially represent a novel manifestation of malathion toxicity, it seems more likely that there was some other unidentified inhaled toxin or that the disease was due to an unidentified micro-organism or fulminant autoimmune process.

We hypothesise this case is rare as this injury would have been lethal in the pre-ECMO era, and that the multiplicity of stenoses here reflects the severity of the original airway injury. In one sense our case highlights what can be achieved with ECMO but, in another, it is a cautionary tale of the morbid sequelae that may arise after ‘surviving the unsurvivable’.

## Data Availability

All relevant data for this case are shared in this manuscript. Further provision of data will not be contemplated due to the priority of patient confidentiality.

## Abbreviations

Aa gradient: alveolar arterial oxygen gradient
ANCA: antineutrophil cytoplasmic antibody
CT: Computed tomography
ECMO: Extra-corporeal membrane oxygenation
FEV1: Forced expiratory volume in 1 s
FiO_2_: Fraction of inspired oxygen
FVC: Forced vital capacity
PaCO_2_: partial pressure of arterial carbon dioxide
PaO_2_: partial pressure of arterial oxygen
